# 

**Published:** 1892-05

**Authors:** 


### CONTENTS.

ORIGINAL COMMUNICATIONS—

Extirpation of the Rectum for Carcinoma. By J. McFadden Gaston, M. D., Atlanta, Ga. .......... 129

A New Method for the Management of Abortion. By Charles H. Harris, M. D., Cedartown, Ga. .......... 135

CLINICAL REPORTS—

Atlanta Medical College .......... 140

SOCIETY REPORTS—

Georgia State Medical Association .......... 142

Clinical Society of Maryland .......... 157

CORRESPONDENCE—

New York Letter .......... 163

EDITORIAL—

The Grady Memorial Hospital .......... 168

The Forty-third Annual Session of the Medical Association of Georgia .......... 171

SELECTIONS .......... 173-183

MEDICAL ITEMS .......... 184-187

BOOK REVIEWS .......... 183-191

INDEX TO ADVERTISEMENTS .......... xii

ITEMS OF INTEREST .......... xi

